# Preventive role of erythropoietin against aminoglycoside renal toxicity induced nephropathy; current knowledge and new concepts

**DOI:** 10.12861/jrip.2013.10

**Published:** 2013-03-01

**Authors:** Mahmoud Rafieian-Kopaei, Hamid Nasri

**Affiliations:** ^1^Medical Plants Research Center, Shahrekord University of Medical Sciences, Shahrekord, Iran; ^2^Department of Nephrology, Division of Nephropathology, Isfahan University of Medical Sciences, Isfahan, Iran

**Keywords:** Erythropoietin, Amikacin, Nephropathy, Renoprotection

**Implication for health policy/practice/research/medical education:**

Erythropoietin is a promising renoprotective drug to prevent or attenuate tubular damage induced by gentamicin or other nephrotoxic agents which act through the same mechanisms as gentamicin. In this regard, to better understand the preventive properties of erythropoietin, more experimental or clinical studies are suggested.

## Dear editor,

Recently attentions were made toward renoprotective efficiency of erythropoietin against renal tubular toxicity of acute kidney injury beyond stimulating erythropoiesis. Kaynar and colleagues, studied the role of erythropoietin (EPO) in prevention of amikacin-induced nephropathy, to explore whether erythropoietin was renoprotective in amikacin-induced nephropathy in a rat model. In their study twenty-eight rats were designated equally into 4 groups. Of them, there was a group in which the rats pretreated with EPO and amikacin. Twenty-four hours after the last injection, renal tissues were excised for histopathological examinations, and blood samples were collected for serum creatinine and blood urea nitrogen measurements. The most severe and pronounced injuries based on tubular necrosis were observed in the amikacin group, while rats pretreated with EPO demonstrated marked reduction of the histological features of renal injury. They suggested a protective effect of exogenous EPO against experimental amikacin-induced renal injury (1). We congratulate for their work, however, we would like to remind a few points about protective role of EPO in aminoglycosid–tubular toxicity. Amikacin belongs to the aminoglycoside antibiotics and have a nephrotoxicity like gentamicin (GM) (1). In an investigation to find out the ameliorative effects of EPO, we studied 40 male Wistar rats with a weight range of 200-250 g (2). They were allocated randomly into 4 groups (10 rats in each). Likewise to the study of Kaynar *et al*., we had a group, in which rats were injected intraperitoneally with a combination of GM (100mg/kg) and EPO 100U/kg intraperitoneally for 10 days. However, in comparison to the study of Kaynar *et al*., there was another group, in which the rats received GM (100mg/kg) for 10 days, then EPO 100U/kg was injected intraperitoneally for the next 10 days and then the rats were sacrificed at the day 20^th^ and kidneys were removed. All specimens were examined for six morphologic parameters including epithelial cell vacuolization, degeneration, tubular cell flattening, hyaline cast, tubular dilatation and debris materials in tubular lumen on a semi-quantitative score from 1 to 5. The score of zero was assigned to the normal tissue without damage. Similarly, the results indicated that, EPO prevented the increase in serum creatinine and blood urea nitrogen. The effect of EPO on damage score, showed that co-administration of GM and EPO, in the two mentioned groups reduced effectively the kidney tissue damage compared to control group (p<0.05). Our study showed the renoprotective effect of EPO, when the drug is given in combination with GM. Moreover, the protective effect of EPO was observed when the drug was applied after GM–induced tubular damage and was revealed that the drug was still effective after installation of tissue damage (2). This indicates that EPO may have curative effect, other than preventive property. We also recently showed the protective effect of EPO against cisplatin-induced nephrotoxicity (3). Thus, EPO is a promising renoprotective drug to prevent or attenuate tubular damage induced by GM or other nephrotoxic agents which act through the same mechanisms as GM (1-3). In this regard, to better understand the preventive properties of EPO, more experimental or clinical studies are suggested.

## Authors’ Contributions

HN wrote the manuscript .MRK contributed to the final preparation of the manuscript.

## Conflict of interests

The author declared no competing interests.

## Ethical considerations

Ethical issues (including plagiarism, data fabrication, double publication) have been completely observed by the author.

## Funding/Support

None.
